# Maternal-Fetal Implications of SARS CoV-2 Infection during Pregnancy, Viral, Serological Analyses of Placenta and Cord Blood

**DOI:** 10.3390/ijerph19042105

**Published:** 2022-02-13

**Authors:** Souhail Alouini, Jerôme Guinard, Olivier Belin, Louis Mesnard, Evelyne Werner, Thierry Prazuck, Chantal Pichon

**Affiliations:** 1 Department of Obstetrics and Gynecology, Centre Hospitalier Régional d’Orléans, 45100 Orléans, France; louis.mesnard@chr-orleans.fr; 2 Faculté des Sciences, Université d’Orleans, 45100 Orleans, France; 3 Department of Microbiology, Virology and Parasitology, Centre Hospitalier Régional d’Orléans, 45100 Orléans, France; jerome.guinard@chr-orleans.fr; 4 Department of Anesthesiology and Reanimation, Centre Hospitalier Régional d’Orléans, 45100 Orléans, France; olivier.belin@chr-orleans.fr; 5 Department of Pediatrics and Neonatal Intensive Unit Care, Centre Hospitalier Régional d’Orléans, 45100 Orléans, France; evelyne.werner@chr-orleans.fr; 6 Department of Infectious and Tropical Diseases, Centre Hospitalier Régional d’Orléans, 45100 Orléans, France; thierry.prazuck@chr-orleans.fr; 7 Centre de Biophysique Moléculaire, CNRS, Université d’Orléans, 45100 Orléans, France; chantal.pichon@univ-orleans.fr

**Keywords:** COVID-19, SARS-CoV-2, SARS-CoV-2 antibodies, coronavirus, cord blood, pregnancy, maternal–fetal transmission, real time-PCR, placenta

## Abstract

Objective: There are few data on the maternal–fetal transmission of SARS-CoV-2 and its outcomes. This study aimed to evaluate pregnancy outcomes of pregnant women infected by SARS-CoV-2, to detect SARS-CoV-2 in placenta and different newborns’ samples and search antibodies in cord blood. Methods: This was a prospective study of pregnant women diagnosed with SARS-CoV-2 infection from May 2020 to May 2021. At delivery, the placentas were investigated for SARS-CoV-2 using RT-PCR, cord blood. Mothers’ blood samples were tested by SARS-CoV-2 serology. PCR of nasopharyngeal, anal and gastric swabs (NPSs) of newborns was performed according to pediatric indications. Results: Among 3626 pregnant women presenting at maternity to deliver, 45 mothers had COVID-19 during their pregnancy or at delivery (32 ± 4.8 years). Most of them were multiparous and in the third trimester. There were 35 (77%) women who remained in ambulatory, while 10 (22%) were hospitalized for severe pneumonia, digestive symptoms, and/or fetal tachycardia. Thirty-eight delivered vaginally, and 7 had a cesarean delivery with normal Apgar scores (9 ± 1.6 at 5 min) and umbilical artery pH (7.22 ± 0.08). Two mothers required ICU admission after cesarean section for fetal and maternal distress. Of the 46 newborns, 6 were premature births (13%) and 5 IUGR (intra-uterine growth restriction,11%). RT-PCR SARS-CoV-2 was positive for 1/30 placental, and 1/33 neonatal anal swabs and negative in all other cases and in gastric swabs. SARS-CoV-2 IgG was positive in 20/41 cord blood samples (49%) and their mothers’ samples. IgM was negative in the 23 cord blood samples. Conclusions: Pregnancy outcomes in women diagnosed with COVID-19 during their pregnancy were favorable in most cases. However, some women with severe clinical forms required hospitalization and ICU admission. Preterm births and intrauterine growth retardations were relatively frequent. Vaginal delivery was possible in most cases. SARS-CoV-2 IgG antibodies were positive and elevated in most cord blood samples of newborns. They are possibly of maternal origin, suggesting a probable mechanism of fetal protection against SARS-CoV-2 infection. No SARS-CoV-2 IgM was found in the cord blood samples. Detection of SARS-CoV-2 in placenta is rare.

## 1. Introduction

The COVID-19 pandemic is responsible for severe acute respiratory syndrome leading to thousands of deaths daily [1]. With almost no age spared, the SARS-CoV-2 infection has been reported in both pregnant and non-pregnant women equally [2,3]. However, the potential mechanism for in utero maternal–fetal transmission of the SARS-CoV-2 virus is uncertain and debatable. As newborns are also known to be infected by the SARS-CoV-2 virus [4], it is important to understand how the virus is transmitted in utero and/or in the postpartum period.

While some studies document that the SARS-CoV-2 was not recovered from the placenta or nasopharyngeal swabs of neonates born to COVID-19 positive mothers [2], a case of elevated SARS-CoV-2 IgM antibody in the neonate’s blood at 2 h of life has been reported [5].

Establishing if there is in utero fetal transmission is important for identifying potential fetopathies that can be treated. In addition, unlike other viral infections (such as cytomegalovirus), it is still undefined whether the natural evolution of COVID-19 in pregnant women has serious consequences for the baby, both in utero and after birth [6,7,8]. Vivanti et al. [9] reported one case of maternal–fetal transmission of SARS-CoV-2. Thus, there is a need to ascertain the possibility of maternal–fetal transmission of COVID-19.

Pregnant women with COVID-19 commonly develop a moderate clinical form of the disease [4]. Likewise, there is a lack of evidence and evidence-based guidelines for fetal well-being and fetal outcomes in the case of maternal COVID-19 infection. In the previous studies reporting on COVID-19-positive pregnant women, the mode of delivery was mainly a cesarean section [2]. However, management of the newborns of mothers infected by COVID-19 remains problematic because of lack of evidence and guidelines in the domain.

Even though few data are available on SARS-CoV-2 serology in the cord blood and the maternal blood, the need for questioning on a possible passive-immunity mechanism in utero transferred by the mother to her fetus remains acute.

Based on these findings, we aimed to evaluate pregnancy outcomes of pregnant women infected by SARS-CoV-2, to detect SARS-CoV-2 in placenta and different newborns’ samples and search antibodies in cord blood.

## 2. Methods

Pregnant women presenting for delivery at the Regional Hospital Center, Orleans, France, with COVID-19 infection from 5 May 2020 to 1 May 2021 were included in the study. The Maternity Department at the Regional Hospital Center, Orleans, France, is a tertiary referral center performing approximately 4900 deliveries per year, while the Hospital’s Department of Infectious Diseases and the Laboratory of Virology are also referral departments for COVID-19. At the beginning of the COVID-19 pandemic in France, we prospectively elaborated a multidisciplinary protocol with infectiologists, virologists, pharmacologists, anesthesiologists, and gynecologists to manage pregnant women coming to the hospital who were suspected of COVID-19 infection 

All pregnant women suspected of SARS-CoV-2 infection based on clinical signs (cough, fever, dyspnea, ageusia, anosmia, digestive symptoms or flu-like syndrome) had a polymerase chain reaction (RT-PCR) SARS-CoV-2 test on the nasopharyngeal swab and/or SARS-CoV-2 serology [10,11,12]. Only women who tested positive for SARS-CoV-2 who were between the ages of 18 and 49 years were included in the study. Written informed consent was obtained from each enrolled patient. S.A., O.B., and L.M. conducted the research. Only pregnant women with SARS-CoV-2 infection during their pregnancy or at delivery who came to the maternity for delivery were included in the study. Midwives, nurses, and doctors were involved in taking the nasopharyngeal swabs of the mothers and their newborns, anal and gastric swabs of the newborns, and of cord blood and placenta samples. All these samples were taken in the delivery room.

The study was approved by the CPP (Committee of Protection of Persons) Ouest II, France: number 2020-A01110-39.

## 3. Biological Exams

The Real Time Polymerase chain reaction (RT-PCR) for SARS-CoV-2 was performed using the TaqPath multiplex COVID-19 kit (Thermo Fischer Scientifc, Waltham, MA, USA). All samples were processed at the Department of Virology, Regional Hospital Center of Orleans, France.

SARS-CoV-2 serology was performed with an immuno-enzymatic technique by chemiluminescence (Maglumi 800). The sensitivity of this technique is 95% at 15 days after the onset of clinical symptoms, and the specificity for IgM and IgG is 99% [13]. The results for SARS-CoV-2 serology were classified as follows:

For IgG: CoV-2 index negative if <0.8; positive if >1.2; and 0.8 < gray zone < 1.2.

For IgM: CoV-2 index negative if <0.03; positive if >0.365.

## 4. Study Sample

The sample consisted of pregnant women who were diagnosed with COVID-19 infection and consented to participate, and prospectively offered a placental and cord blood sample at delivery. RT- PCR for SARS-CoV-2 was done for the placental tissue, and SARS-CoV-2 serology was performed on the cord blood. According to pediatric indications, anal and gastric samples of newborns were analyzed by PCR. In case of positive anal or gastric swabs for SARS-CoV-2 of the newborn, a PCR on vaginal and anal swabs of the mother was performed. In the case of respiratory or digestive clinical symptoms in the newborn or perinatal SARS-CoV-2 infection by the mother, a PCR on the neonate’s nasopharyngeal swabs was performed, according to the pediatric indications. All pregnant women suspected of COVID-19 or with a history of COVID-19 during their pregnancy had serum examination for SARS-CoV-2 at the time of admission to the maternity center. All these samples were collected immediately after delivery in the operating room.

Besides the patients’ demographic characteristics (age, BMI, parity, geographic origin), data for the number of weeks of gestation (WG), previous medical and obstetric history, comorbidities, clinical symptoms, the results for SARS-CoV-2 PCR and/or SARS-CoV-2 serology for pregnant women COVID-19 were recorded.

Severe COVID-19 was defined as dyspnea, respiratory rate of ≥30 breaths per min, blood oxygen saturation <94%, lung infiltrates >50%, or need of mechanical ventilation or multiple organ failure [14,15].

For fetal monitoring, the outcomes such as delivery mode, birth weight, Apgar scores, and umbilical artery pH were recorded for analysis. The following maternal outcomes were noted: hospital stay duration, follow-up for ambulatory patients, clinical and biological follow-up.

## 5. Statistical Analysis

Descriptive statistics, either absolute numbers and percentages for qualitative data or mean and standard deviations for quantitative data, were calculated. Followed by descriptive analyses using MS-Excel version 16.16.21 (Microsoft Corporation, Redmond, WA, USA).

## 6. Results

Among the 3626 patients presenting to the department of Obstetrics to deliver during the study period, 45 pregnant women (with a mean age of 32 ± 4.8 years) diagnosed with COVID-19 during their pregnancy and 46 newborns (1 twin pregnancy) were included in the study. Most of them were multiparous and in the third trimester of pregnancy (Table 1).

All pregnant women who came to the hospital for delivery were tested for COVID-19. The RT-PCR test on nasopharyngeal swabs was positive for all 45 patients, confirming the diagnosis of COVID-19 infection. No pregnant women with covid-19 were vaccinated during the study period. The major symptoms were fever, cough, ageusia, headache, dyspnea, anosmia, diarrhea or vomiting, and asthenia (Table 2). Most women were followed ambulatory. However, ten pregnant women (22%) required hospital admission for major symptoms or in the case of fetal distress (Table 3).

Of the 45 women included in the study, 38 delivered vaginally (84.4%) and 7 (15.6%) by cesarean delivery (CD). Tenofovir was prescribed at the beginning of the study to 4 patients. Two women were admitted to the intensive care unit (ICU) and required mechanical ventilation for 30–45 days after cesarean delivery for severe COVID-19. The two patients were older than the others (age > 40 years). One of them presented with severe anemia and the other with previous phlebitis and diabetes. Both also presented with severe pneumonia. The mean interval between the COVID-19 infection and delivery was 48 ± 53 days, varying from 0 to 220 days (Table 4). The mean umbilical artery pH was 7.22 ± 0.08, while Apgar scores were within normal limits (9 ± 1.6). There was one twin pregnancy. Of the 46 newborns, 6 were premature births (13%), 5 IUGR (intra-uterine growth restriction,11%), and 5 had macrosomia (>90th percentile).

SARS-CoV-2 was detected by RT-PCR in 1(3.3%) of the 30 placental samples at delivery (Table 5).

The PCR of the placenta did not detect SARS-CoV-2 in the 29 other cases.

SARS-CoV-2 was detected by RT-PCR in 1 (3%) out of 33 anal swabs of newborns taken immediately after delivery. These results were verified by RT-PCR of the same samples after two days, which remained positive.

PCR was negative in the maternal vaginal and anal swabs. The RT-PCR on NPS of the newborn was also negative at day 2.

RT-PCR on newborn rectal and gastric samples was negative in all other cases (see Table 4), and all 13 NPSs of newborns suspected of COVID-19 were negative on RT-PCR and were healthy.

The IgG antibodies for SARS-CoV-2 were positive in 20 of the 41 cord blood samples (48.8%) (Table 5), while IgM in the cord blood was negative for all cases (23/23). Likewise, IgG in the maternal blood was positive in 20 of the 37 cases (54%) and IgM was positive in 4 of the 23 cases (17%).

It was not possible to collect amniotic fluid samples because most women delivered vaginally and many of them had a premature or spontaneous rupture of membranes mixing the amniotic fluid with vaginal fluid. However, the rectal and gastric swabs were collected to reflect the amniotic status.

## 7. Discussion

Forty-five mothers at delivery were included in this prospective study. The sample is considered small in clinical; however, it should be noted that clinics were not the main focus of the study. In the study period, we did not observe a high number of pregnant women infected by SARS-CoV-2 who delivered, even though our maternity is among the biggest in France. The aim of the study was more focused on the biological investigation of the maternal–fetal transmission of SARS-CoV-2 by analyzing cord blood and placental samples. Our study is prospective and one of the largest studies concerning the number of cord blood, placental samples, and anal and gastric swabs analyzed. Cosma et al. [16] analyzed 17 pregnant women who tested positive for COVID-19 and found that their newborns developed IgG antibodies. Colson et al. [17] analyzed 31 placentas of mothers who tested positive for COVID-19 and found one case of placental infection with SARS-CoV-2.

The 45 pregnant women with COVID-19 who participated in this study experienced clinical symptoms that varied greatly, from mildly symptomatic to severe forms that required hospitalization. COVID-19 was more easily detected in the last trimester of pregnancy; perhaps the infection was less symptomatic in the first trimester of pregnancy and thus less susceptible to detection. Most patients were managed symptomatically and were given ambulatory care. Those who were hospitalized developed more severe symptoms of COVID-19 and experienced acute respiratory signs, such as moderate-to-severe pneumonia, with or without digestive symptoms of diarrhea, vomiting, and nausea. Ellington et al. [2] found that pregnant women were likelier to be hospitalized and require mechanical ventilation compared to non-pregnant women. As for the present study, only two patients needed mechanical ventilation. Our study showed that COVID-19 in pregnant women manifested mainly in benign forms, even though some patients needed mechanical ventilation, a result that differed from initial studies, which reported that COVID-19 was merely a benign disease in pregnant women. Despite the low number of patients hospitalized in the ICU, the findings are in accordance with other studies, which highlighted that the majority of pregnant women COVID-19 experienced minor or moderate forms of the disease. [18].

Most pregnant women with COVID-19 delivered a healthy newborn at term vaginally. Premature birth was frequent, and CD was indicated for 15% of women. Previous reports [2] have documented cesarean delivery as most frequently used in COVID-19 patients. In the current study, vaginal delivery has proven to be safe and should be emphasized for pregnant women with COVID-19.

In our study, SARS-CoV-2 was detected in one placental sample and one anal swab of a neonate (born to a mother with a history of COVID-19 during pregnancy) taken immediately after delivery. RT PCR SARS-CoV-2 of NP swabs and anal swabs of their mothers were negative at delivery. Vivanti et al. [9] reported a case of maternal–fetal transmission of SARS-CoV-2. They detected the virus in the placenta. Hence, the presence of SARS-CoV-2 in the placental and anal samples of a newborn immediately at birth suggests maternal–fetal transmission of SARS-CoV-2 during pregnancy or a maternal contamination of samples at delivery.

According to Shah et al. [19], when SARS CoV-2 is not found in the cord blood and the placenta, the maternal–fetal transmission is unlikely. However, Gong et al. [20] reported positive anal swabs in 10 children, even with negative throat swabs. Feco–oral transmission is also a probable route for SARS-CoV-2 infection as suggested by Yuan et al. [20], who found SARS CoV-2 in anal swabs of children with COVID-19 infection.

It is noteworthy that Chen et al. [4] did not find viral RNA in 3 placental tissues obtained from women with COVID-19 infection. Similarly, Fan et al. [8] reported that SARS-CoV-2 was not detected by PCR in two products of conception and infants. In the same way, Chen et al. [2] have reported that the amniotic fluid and cord blood in 6 infected patients was negative for SARS-CoV-2 on PCR. However, the placenta, anal, and gastric samples of the newborns were not tested, and no serodiagnosis of SARS-CoV-2 was performed on the cord blood [2] in the placenta, anal and gastric, and nasopharyngeal samples of the neonates born to COVID-19-positive mothers.

In this study, SARS-CoV-2 IgG antibody levels were elevated in cord blood samples at delivery, as well as in mothers’ blood. The presence of IgG in the cord blood of many newborns is probably due to the passage of maternal IgG antibodies in the cord blood. These antibodies are probably protective against COVID-19 infection in the fetus and may explain why the fetus is rarely infected by the virus. In many cases, the SARS-CoV-2 IgG serology was negative in both of the aforementioned samples. In these cases, the mother was asymptomatic, indicative of a low viral charge. In addition, many COVID-19 mothers did not develop IgM and IgG antibodies even in the presence of a positive PCR of their nasopharyngeal swabs. This is probably due to low viral-plasmatic circulation.

Another important finding was that we did not discover SARS-CoV-2 IgM antibodies in all cord blood samples tested, even though the sensitivity and specificity of the immune-enzymatic technique used were excellent at 95% and 99%, respectively. However, Dong et al. [5] reported one case of elevated IgM antibodies at two hours of life in a newborn.

## 8. Limitations and Strengths of the Study

First, the sample size was relatively small; however, this prospective investigation is among the largest studies that have evaluated the maternal–fetal transmission of SARS-CoV-2. The study includes prospective analyses and the results of placental, cord blood samples, and anal, gastric, and nasopharyngeal swabs of newborns and COVID-19 mothers at delivery.

Second, not all samples could be tested, as our hospital was overwhelmed by the COVID-19 waves. At times, the samples did not arrive at the laboratory in good condition. In other cases, the blood samples were hemolyzed; it was then impossible to perform viral analysis.

In addition, our study’s strength is that it demonstrated that vaginal delivery of healthy newborns of mothers with COVID-19 is possible in the majority of cases; this contrasts with the findings of other studies, wherein caesarean section deliveries were performed in most cases [2].

Our study is among the largest studies to have prospectively analyzed (contrary to many retrospective studies) placenta and cord blood samples. It showed that pregnant women with COVID-19 transmitted SARS-CoV-2 IgG antibodies to their fetuses via cord blood to protect them in utero against an acute fetal viral infection. These findings provide a probable explanation for why very few fetuses were infected in utero by SARS-CoV-2 and are correlated with the fact that we did not find SARS-CoV-2 IgM in the analyzed cord blood samples. Indeed, the presence of IgM in the blood signifies an acute infection in utero. Other authors found IgM in the cord blood in one case [5]. Thirty placentas were analyzed prospectively for SARS-CoV-2 at delivery. This sample is among the largest studies that have analyzed the presence of the virus in the placenta. It showed one case of a placenta positive for SARS-CoV-2, which suggests in utero SARS-CoV-2 transmission. This study confirms the results of other studies: maternal–fetal transmission of SARS-CoV-2 is possible, but it remains rare.

The analysis of both placental and cord blood samples explains why in utero viral infection is rare.

## 9. Conclusions

In this prospective study, most women recovered well with symptomatic management. However, patients with severe clinical forms required hospitalization or ICU admission. There were preterm births and intrauterine growth restrictions in pregnancies complicated by COVID-19. Pregnant women with COVID-19 should be carefully monitored clinically and by ultrasonography in order to diagnose these pathologies.

Vaginal delivery was possible and should be recommended for the majority of pregnant women with COVID-19.

Our study showed that pregnant women with COVID-19 transmitted the SARS-CoV-2 Ig G antibodies to their fetuses via the cord blood. These findings could be a very probable explanation of why very few fetuses are infected in utero by SARS-CoV-2 and are correlated to the fact that we did not find the SARS-CoV-2 IgM in the analyzed cord blood samples. These findings suggest a probable mechanism of fetal protection and passive fetal immunization against SARS-CoV-2 infection.

We found one case of positive placenta for SARS-CoV-2, which suggests in utero SARS-CoV-2 transmission. Future larger studies are highlighted to confirm our results.

## Figures and Tables

**Table 1:** General characteristics of pregnant women with coronavirus disease (COVID-19).

Characteristics	N	%
Age, mean ± SD, years	32 ± 4.8	
Multiparous	31	68.89
Weeks of gestation	31.14 ± 8.25	
mean ± SD at diagnosis		
Trimester of pregnancy with COVID-19		
1st trimester	4	8.89
2nd trimester	3	6.67
3rd Trimester	38	84.44
Ethnic group		
White	36	80.00
Black	9	20.00
Comorbidities		
BMI (kg/m²) > 30	8	17.78
Gestational diabetes	10	22.22
Hypothyroidism	4	8.89
Severe eczema	1	2.22
Asthma	1	2.22
Age > 35 years	11	24.44
Previous phlebitis	1	2.22
Anemia < 8 g Hb/dL	5	11.11

**Table 2:** Clinical symptoms of pregnant women diagnosed with COVID-19.

Mother Characteristics	No.	Percent (%)
Clinical Symptoms COVID-19 (n = 45)	26	57.78
Cough	11	24.44
Fever	17	37.78
Dyspnea	9	20.00
Flu-like syndrome	15	33.33
Diarrhea/vomiting	8	17.78
Ageusia	10	22.22
Anosmia	8	17.78
Asymptomatic	19	42.22

**Table 3:** Diagnosis and outcomes of pregnant women with coronavirus disease (COVID-19).

Mother Characteristics	No.	Percent (%)
COVID-19 Diagnosis of the Mother (*n* = 45)		
PCR NP swabs +	45	100.00
Followed ambulatory	35	77.78
Hospitalized	10	22.22
Pneumonia	6	13.33
Moderate	3	6.67
Severe	3	6.67
Pharmacotherapy		
Paracetamol	17	37.78
Antiviral treatments	4	8.89
Heparin treatment	15	0.30
Oxygen therapy	8	17.78
Intensive unit care	2	4.44

**Table 4:** Delivery of mothers COVID-19 and newborns outcomes.

	Outcomes/N	Standard Deviation
Mean day interval of COVID-19 delivery (days)	48	53
Term of birth (weeks of gestation)	38.7	1.7
Caesarean Delivery	7	
Vaginal delivery	38	
Birth weight (g)	3154.4	905.5
Apgar score at 3 mm	9.4	1.6
Umbilical artery pH	7.22	0.08

**Table 5:** SARS-CoV-2 findings at delivery in the placenta, cord blood, and newborns of mothers with COVID-19.

PCR/Serology SARS-CoV-2	Total	*n* Positive	Positive %
IgG of mother at delivery	37	20	54.05
IgM SARS-CoV-2 of mother	23	4	17.39
PCR SARS-CoV-2 placenta	30	1	3.33
IgM cord blood	23	0	0.00
IgG cord blood	41	20	48.78
Anal newborn PCR swabs	33	1	3.03
Gastric NB PCR swabs	33	0	0.00
Nasopharyngeal newborn PCR	13	0	0

## Data Availability

Data are available from the corresponding author under reasonable request.
